# A Deficit in Face-Voice Integration in Developing Vervet Monkeys Exposed to Ethanol during Gestation

**DOI:** 10.1371/journal.pone.0114100

**Published:** 2014-12-03

**Authors:** Shahin Zangenehpour, Pasha Javadi, Frank R. Ervin, Roberta M. Palmour, Maurice Ptito

**Affiliations:** 1 Department of Psychology, McGill University, Montreal, QC, Canada; 2 School of Optometry, University of Montreal, Montreal, QC, Canada; 3 Department of Psychiatry, McGill University, Montreal, QC, Canada; 4 Department of Human Genetics, McGill University, Montreal, QC, Canada; 5 BRAINlab, Department of Neuroscience and Pharmacology, University of Copenhagen, Copenhagen, Denmark; 6 Laboratory of Neuropsychiatry, Psychiatric Centre Copenhagen and Department of Neuroscience and Pharmacology, University of Copenhagen, DK-2100, Copenhagen, Denmark; Université de Montréal, Canada

## Abstract

Children with fetal alcohol spectrum disorders display behavioural and intellectual impairments that strongly implicate dysfunction within the frontal cortex. Deficits in social behaviour and cognition are amongst the most pervasive outcomes of prenatal ethanol exposure. Our naturalistic vervet monkey model of fetal alcohol exposure (FAE) provides an unparalleled opportunity to study the neurobehavioral outcomes of prenatal ethanol exposure in a controlled experimental setting. Recent work has revealed a significant reduction of the neuronal population in the frontal lobes of these monkeys. We used an intersensory matching procedure to investigate audiovisual perception of socially relevant stimuli in young FAE vervet monkeys. Here we show a domain-specific deficit in audiovisual integration of socially relevant stimuli. When FAE monkeys were shown a pair of side-by-side videos of a monkey concurrently presenting two different calls along with a single audio track matching the content of one of the calls, they were not able to match the correct video to the single audio track. This was manifest by their average looking time being equally spent towards both the matching and non-matching videos. However, a group of normally developing monkeys exhibited a significant preference for the non-matching video. This inability to integrate and thereby discriminate audiovisual stimuli was confined to the integration of faces and voices as revealed by the monkeys' ability to match a dynamic face to a complex tone or a black-and-white checkerboard to a pure tone, presumably based on duration and/or onset-offset synchrony. Together, these results suggest that prenatal ethanol exposure negatively affects a specific domain of audiovisual integration. This deficit is confined to the integration of information that is presented by the face and the voice and does not affect more elementary aspects of sensory integration.

## Introduction

Exposure to ethanol in utero is recognized as a leading cause of preventable developmental disorder [1]. The continuum of fetal alcohol spectrum disorders (FASD) ranges from craniofacial dysmorphology and mental retardation to the much more common developmental learning and behavioural disorders which are now reaching epidemic proportions [2], [3]. Many of the neurobehavioral deficits may not be apparent until the educational years [4] and are typically not recognized as a result of fetal alcohol exposure. This prevalence may be perpetuated, in part, by a failure to diagnose FASD in the clinical setting [5], [6], and this in turn may contribute to a public misconception that moderate prenatal alcohol exposure, particularly during the last trimester, has relatively little impact on the developing foetus [7].

The primary focus of FASD clinical studies has traditionally been on ethanol-induced deficits in social competence [8], executive function [9], cognition [10], attention [11], and memory [8]. Problems in social cognition are both common and of particular relevance to the behavioural outcome of Fetal Alcohol Exposure (FAE) [12]. Children with FAE often have difficulty perceiving social cues and are likely to exercise poor judgment in social situations, which impedes their ability to behave in socially appropriate ways or maintain successful peer relationships [8], [13], [14]. In addition, studies of animal models of FASD have revealed significant deficits in social behaviour ranging from behavioural alterations in maternal-infant dyad [15]–[17] during infancy and early postnatal development, to changes in active social interactions [18] and increased aggression in males [19], [20] during late adolescence and adulthood.

Social behaviour is a complex phenomenon, which depends on the intricate interactions amongst genetic factors, neurobiological development, early life experiences related to socialization, and learning throughout one's lifespan. Behaviour is generally considered to be the outcome of the sequence of sensation, perception and cognition. Therefore, it stands to reason that some of the overt social behavioural abnormalities associated with FASD stem from deficits in the sensory and perceptual processing of socially relevant information, as supported by recent studies on sensory-specific deficits observed in children with FASD [21]–[23].

In turn, socially relevant signals in primates for the most part contain information originating from faces and voices of conspecifics. Therefore, perception of social cues relies heavily on the proper integration of auditory and visual stimuli containing corresponding facial and vocal information, which contain communicative and/or affective content. It is now well documented that perception of faces [24]–[26] and voices [27]–[29] is mediated through various regions of the temporal lobes with both modality-specific and multisensory functions. In addition, integration of those modality-specific representations of socially relevant cues has recently been documented to take place in various regions of the frontal lobes [30]–[34]. Therefore, these findings suggest that the integrity of the frontal lobes may necessarily contribute to the proper integration of facial and vocal information to give rise to veridical representations of socially relevant cues.

Magnetic resonance imaging of FASD patients suggests an overall reduction in brain size with a disproportionate reduction found only in the parietal lobe [35]. It has also been shown in a gestational study that the volume of the frontal cortex is inversely correlated with maternal alcohol consumption [36]. Ervin and colleagues [37], [38] have developed a naturalistic non-human primate model of FASD. Burke and colleagues [39] have reported that alcohol-exposed monkeys approximately equivalent to 8-year-old humans have a 35% lower neuronal population in the frontal lobes without a significant alteration in total cortical volume. This was the first study to show that prenatal ethanol exposure during the third trimester, at sub-intoxicant doses, resulted in a lowering of the total neuronal population in the area commonly associated with the neurobehavioral deficits observed in children with FASD: namely, the frontal lobes.

Three converging lines of evidence thus formed the foundation of the current work: i) pervasive social cognitive deficits documented in FASD both in humans and animal models; ii) the pivotal role that the frontal lobes play in the integration of socially relevant audiovisual cues emerging from the processing of facial and vocal information; and iii) recently documented significant neuronal reduction in the frontal lobes of vervet monkeys with prenatal ethanol exposure. We therefore reasoned that one of the ways in which the previously reported frontal lobe neuronal reduction may manifest itself may be in the form of a deficit in the integration of socially relevant cues. As such, we tested the hypothesis that, unlike their normally developing counterparts, vervet monkeys with prenatal ethanol exposure will exhibit a deficit in the multisensory integration of socially relevant cues. We tested this hypothesis by using the intersensory paired-preference procedure which has been previously used to study sensory integration capacities of normally developing human infants [40] and young vervet monkey [41].

## Methods

All animal experiments (behavioural studies) were reviewed and approved by the McGill University Animal Care Committee and the Ethics Subcommittee as well as the research ethics board at the Behavioural Sciences Foundation. All animals, housed and handled in strict accordance with good animal practice under supervision of veterinarians, received environmental enrichment and were monitored for evidence of disease and changes in attitude, appetite, or behavior suggestive of illness. In accordance with the recommendations of Weatherall report, “The use of non-human primates in research,” every effort was made to alleviate animal discomfort and pain by appropriate and routine use of anesthetic and/or analgesic agents.

### Subjects

The subjects consisted of two groups of developing vervet monkeys. In the first experiment, the group consisted of 56 normally developing animals ranging in age between 23 and 65 weeks and a mean age of 37 weeks, and 10 ethanol-exposed animals with ranging in age between 29 and 51 weeks and a mean age of 40 weeks. In the second experiment, the group, chosen from a separate cohort of vervets, consisted of 55 normally developing animals ranging in age between 21 and 50 weeks with a mean age of 31 weeks, and 9 ethanol-exposed (FAE) animals ranging in age between 22 and 71 weeks with a mean age of 34 weeks. Both normally developing subgroups were randomly selected from the offspring of a larger colony of adult vervets maintained at the BSF, while the subgroups consisted of the entire cohort available at the time of testing. The subjects were naïve to the experimental procedures. In addition, these monkeys had never been exposed to other species of primates, except for humans. Table 1 summarizes animal profiles in terms of start, duration and total amount of ethanol exposure in both FAE cohorts, as well as other pertinent data.

**Table 1:** Animals' profile.

Subject	Sex	Alcohol started*	Duration of exposure#	Alcohol/day (g/kg)	Total exposure‡	Age at testing (weeks)
***Cohort 1***						
O2780-4	F	135	30	1.99	59.70	29
O3019-3	F	136	34	2.61	52.24	37
O3066-4	M	100	65	1.75	66.50	27
O3295-3	M	94	71	2.63	107.8	37
O3307-2	F	113	52	2.82	81.70	29
O5011-3	M	77	88	3.09	148.40	39
O5106-1	M	109	56	1.79	59.21	51
O5219-1	M	106	59	2.45	88.36	50
O5332-1	F	106	59	2.19	76.54	44
O5335-2	F	110	55	2.51	90.28	51
***Cohort 2***						
O2556-3	F	65	100	2.65	153.72	36
O3065-7	F	119	46	4.22	118.30	23
O3066-5	F	114	51	2.70	88.83	23
O3082-5	F	65	100	1.92	111.50	34
O3085-5	F	114	51	2.18	67.60	22
O3293-4	F	121	44	3.92	102.00	19
O3327-3	F	119	46	4.15	126.20	22
O5010-3	F	122	43	2.76	66.33	47
O5330-3	F	135	30	3.04	60.86	26

*Gestational day alcohol started computed on the basis of 165 days full-term gestation.

# Duration of exposure calculated as the number of weeks between the start of ethanol exposure and delivery.

‡ Total number of g/kg over the entire exposure period based on the amount the dam drank during pregnancy and her body weight before pregnancy.

### Maternal Ethanol Exposure

Healthy female African green monkeys (*Chlorocebus sabeus*) were screened for voluntary alcohol consumption according to previously published method [42]. Females that voluntarily and reliably drank at least 2 g alcohol/kg in a 4-hour scheduled period were identified and housed socially with alcohol-avoiding male breeders. Females were monitored behaviourally and physically for evidence that pregnancy was initiated, then examined biweekly for timing of gestation.

Beginning at about embryonic day 110 of the normal 165-day gestational period, pregnant females were given access to alcohol or an isocaloric sucrose control mixture on 4 days of the week (M,T,Th,F). Alcohol was prepared as a 10% w/v solution and presented in a calibrated drinking bottle. Tap water was always concurrently available. The alcohol bottle was monitored hourly, and remained available for a 5 h period. At the end of the 5 h drinking period, the drinking bottle was removed and the quantity consumed was recorded. Mothers did not continue to drink after the birth of infants.

Blood (1 ml, saphenous vein) was drawn without anaesthesia at the end of the drinking period during weeks 2, 4, 6 and 8 for the measurement of blood alcohol level (alcohol dehydrogenase method, Sigma). Hematocrit was followed as a measure of general health, and a standard clinical battery of liver and other enzymes was measured prior to any intervention, and after 6 weeks of alcohol exposure.

Animals were housed in the laboratories of Behavioural Sciences Foundation (BSF), St Kitts in enriched social environments. The subjects were fed Harlan Teklad high-protein primate chow (5% body weight per day) and fresh local fruit, with water available ad libitum. The experimental protocol was reviewed and approved by the McGill University Animal Care and Use Committee (Canadian Council on Animal Care) and the St Kitts Institutional Review Board.

All offspring were examined shortly after birth for signs of facial dysmorphology and neurological impairment. Offspring were group-housed with their mothers until the age of 6 months, then lived for another 6 months in a nursery setting.

### Apparatus

An animal technician, blind to the purposes of our experiments, held a subject while seated in the centre of a three-sided enclosure whose sides were covered by a thick dark curtain to isolate the subject from the experimenter. In the middle of this enclosure, a 20-inch Apple Cinema Display presented the video component of the audio-visual stimuli. This LCD panel was connected to an Apple MacBook Pro with a 2.0 GHz Intel Core Duo processor, which was used to deliver the stimuli on a trial-by-trial basis. The audio component of the stimuli was presented using a single speaker connected to the laptop computer. The speaker was placed in the middle and under the LCD panel. A Canon Optura 600 digital video camcorder was mounted on a Manfrotto video tripod and placed in the middle and above the LCD panel in order to provide a live feed of all experiment sessions to the experimenter and to record the looking behaviour of each subject for subsequent off-line analysis.

### Stimuli

**Figure 1:**
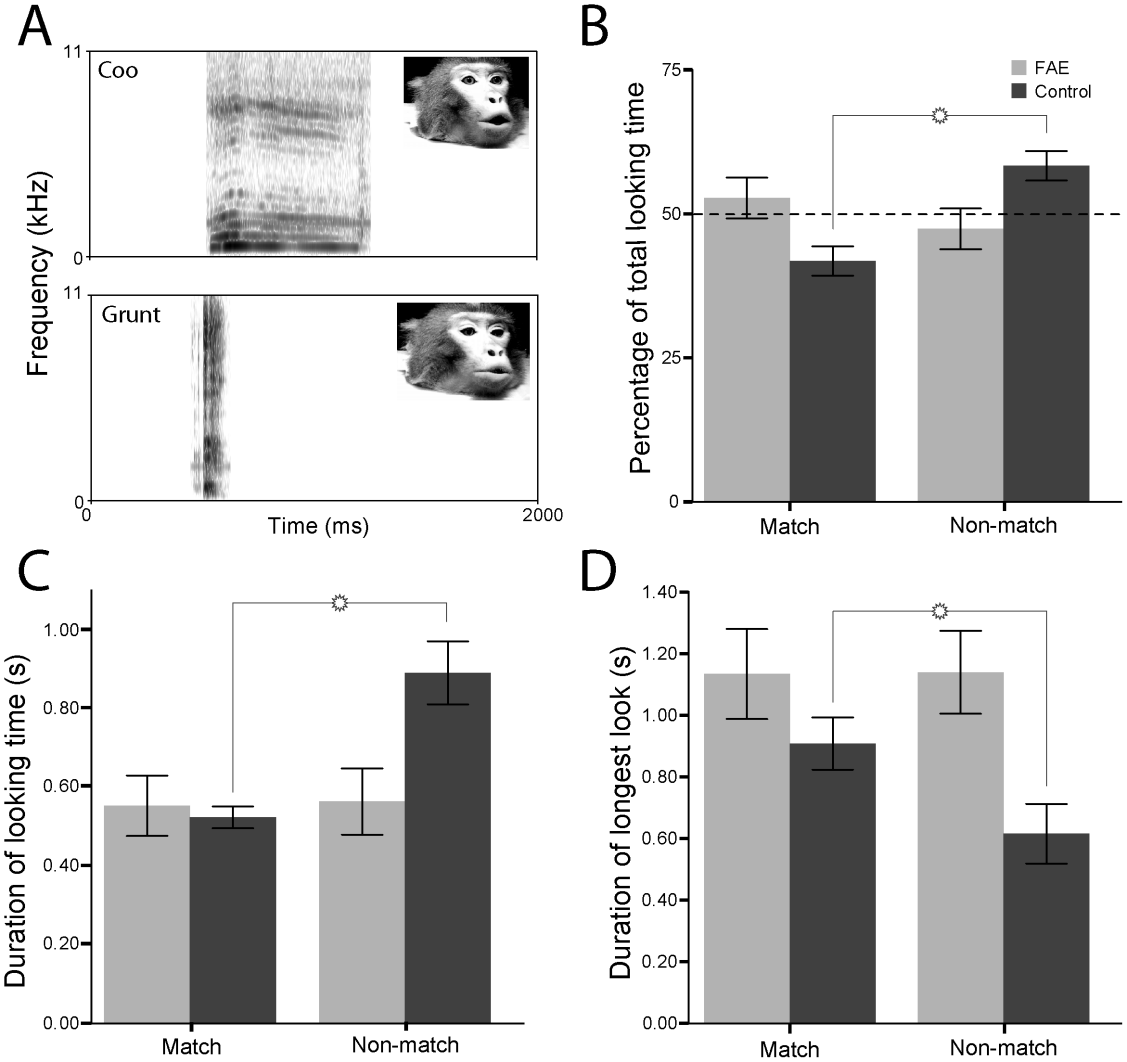
Looking behaviour of control (dark grey) and FAE (light grey) infant vervets at dynamic audiovisual presentations of macaque calls in Experiment 1. A. Spectrograms of the rhesus monkey coo and grunt. Inset shows one frame of the peak of the corresponding facial expression. B. The percentage of total looking time that the subjects spent looking at the matching face. C. The mean duration of looking time at the matching and nonmatching faces. D. The mean duration of the longest single look at the matching and nonmatching faces. Error bars represent ±1 SEM.

We tested the vervets with three different stimulus sets. In Experiment 1 with the first group of vervets, we tested them with the coo and grunt calls of the rhesus monkey (*Macaca mulatta*). Two sets of calls were used and produced by two different rhesus monkeys. Figure 1A shows exemplars of one of the two call pairs we used in this experiment. In addition, Videos S1 and S2 provide samples of the actual face-voice stimuli used to test our subjects. The duration of each video clip containing each call was 2 seconds and the videos were temporally aligned to the onset of mouth movements. It is important to note here that rhesus monkeys look very different from vervet monkeys. The former have a pinkish face surrounded by tan-coloured fur, while the latter have black faces fringed with white fur and then surrounded by a greenish brown fur. In addition, vervet monkeys do not produce coo calls; nearly all Old World monkeys produce a grunt-like call. The coo call was on average 715.3 msec long while the average duration of the grunt call was 142.3 msec based on two macaque callers.

It should also be noted that there is a natural delay for every vocalization (including human speech) between the onset of mouth movements and the onset of vocal sound. This delay can vary considerably (from tens to a few hundred milliseconds) across both different call types and across different exemplars of the same call type. The pattern of reported results reveals that this temporal factor had no impact on intersensory matching. The same is true for previous results in human infants [43], [44] and adult rhesus monkeys [45]. For a description of such face-voice delays in rhesus monkey vocalizations, see [46], [47].

In the second experiment with the second group of vervets, we used two different stimulus sets. In one stimulus set, the vocal component of the stimuli used in Experiment 1 was replaced with a complex tone (triangular waveform, Adobe Audition 1.5) that matched the call's duration but removed any temporal modulation in the envelope of the signal. The fundamental frequency (F0) of the complex tone was based on an average between the fundamental frequencies of the coo and the grunt of one individual.

For both stimulus sets, the pairs of videos were presented side-by-side in the centre of the LCD panel on a black background such that each video frame measured 15.7 cm wide by 10.4 cm high with a horizontal distance of 11.4 cm between the closest edges of the two frames. When a subject looked at the fixation cross located at the centre of the LCD panel at a viewing distance of approximately 40 cm from the LCD panel, each video frame subtended approximately 20° of visual angle on either side of the fovea. The audio track was played at approximately 73 dB sound pressure level measured at the subject's ears.

### Experimental procedures

We used an intersensory paired-preference procedure to determine whether subjects could match the audio signal to the corresponding visual stimulus. A correct match was judged to have occurred if the subject looked longer at the corresponding than the non-corresponding visual stimulus in the presence of the sound than in its absence. In Experiment 1 (i.e., Face + Voice condition), the looking behaviour of each subject was recorded during eight 20-sec trials. A single trial consisted of a 4-sec silent presentation of two calls made by the same macaque monkey (to examine if there is any preference to one of the facial experessions without vice), followed by 16 seconds during which the two videos were presented together with a single audio track matching the content and duration of only one of the two videos. The total of 8 trials ensured that each subject was exposed to all arrangements (i.e., left vs. right), callers (i.e., two macaques), and call types (i.e., coo vs. grunt) for which a match between a video and the audio streams could be made.

In Experiment 2 (i.e., Face + Complex Tone condition and Checkerboard + Tone condition), with two other stimulus sets, the procedure was virtually identical to that of Experiment 1 except that looking behaviour was recorded during twelve 20-sec trials, clustered into four 3-trial groups. Each subset of 3 trials began with a silent trial during which two side-by-side videos of the same macaque monkey mouthing two different calls or two side-by-side different-duration checkerboards were presented repeatedly. This was followed by two trials (counterbalanced for side of presentation) during which subjects saw the same two videos and a single audio track that matched the onset/offset of only one of the two videos.

A coder, who was blind to the testing conditions and to the stimuli being presented on a given trial, measured the direction of looking based on the refelection of both monitors on the corneal surface of monkeys' eyes (i.e., left video frame, right video frame or away from the monitor) and the duration of each look throughout each trial. Inter-observer reliability was computed on a sample of randomly chosen subjects. The average level of agreement on the total duration of looking on each side per trial was 96% for Experiment 1 and 97% for Experiment 2.

### Pupillary response measures

Fifteen normally developing subjects were randomly selected from each of the Face + Voice and Face + Complex Tone conditions, in addition to the entire sample of in each condition (n = 10 and n = 9, respectively). For each subject two measurements of pupil diameter were obtained: one at the end of the longest look at a matching video and the other at the end of longest look at the non-matching video. Pupil diameter measurements (in pixels) were made by first enhancing the contrast of each image in Adobe Photoshop CS4. Then a circular marquee was placed at the boundary that separated the black pupil region from the dark amber color of the iris of the same eye in each captured frame. A ratio of pupil diameter was computed by dividing the measurement in the matching by the nonmatching pupil diameters. In addition, we scored whether, following the single longest look, subjects looked away from both video frames (averted gaze) or looked toward the other video frame.

### Statistical analysis

Data for average looking time and longest looking time, as the main indicators of preferential looking test, were analyzed using analysis of variance (ANOVA). The analyses were performed using SPSS version 21 for Mac OS X with the α level set at 5%.

## Results

The purpose of our study was to determine whether young vervet monkeys with FAE can recognize the correspondence between the faces and voices presented through primate vocalizations, in the same manner that their normally developing counterparts do. To do so, in the first experiment, we compared the amount of looking that vervets accorded to each of two facial expressions made when another monkey was vocalizing two different calls—a coo or a grunt—in the presence of the audio version of one of these calls versus looking at the same faces in silence (Figure 1A; please see the Methods section for more details). Three patterns of looking were possible. First, the subjects could have spent equal amounts of time looking at each face. This would have indicated that they did not detect any correspondences across modalities. Second, they could have spent a greater proportion of time looking at the matching face. This is the typical result that is obtained in human infant studies and adult monkey studies [48]–[52]. Finally, subjects could have spent a greater proportion of time looking at the non-matching face. This outcome would be in line with the response of normally developing young vervet [41].

We tested FAE vervets from 27 to 51 weeks of age (n = 10) and normally developing vervets from 23 to 65 weeks of age (n = 56). To make sure that our results were not affected by the facial identity of a specific monkey presented during testing, the particular voice presented, and presentation side of a particular facial expression, we analyzed the Looking Time data with an ANOVA for repeated mesaures with Caller (i.e., identity of the macaque presenter), Call (i.e., coo vs. grunt) and Side (i.e., the left or the right side of the video screen) as the within-subjects factors. This analysis yielded neither any significant interactions between factor nor any main effects. Therefore, seeing that there were no systematic biases towards any of those factors, the data were collapsed across all those factors and analyzed further (ANOVA) for detecting significant differences based on looking time towards matching and non-matching videos. The second ANOVA for repeated measures was conducted with Group (FAE vs. Control) as the between-subjects and Condition (Match vs. Non-match) as the within-subjects factor for analyzing the proportion of looking time. The analysis revealed a Group × Condition interaction (p = 0.028). The subjects in the control group spent a greater proportion of their total looking time (in %) at the non-matching face than at the matching face while FAE monkeys spent their looking time equally toward the matching and non-matching videos (Figure 1B). In addition, running the same analysis on average looking time (in msec) revealed both a main effect of Condition (p = 0.018) as well as a Group × Condition interaction (p = 0.041). Once again, the subjects in the control group were found to spend a greater amount (in msec) of their total looking time at the non-matching face than at the matching face while FAE monkeys spent their looking time equally toward the matching and non-matching videos (Figure 1C). Lastly, we also measured another index of intersensory matching: the longest single look. Other studies [48], [53], [54] have found that the longest look also provides a useful measure of intersensory integration.

Overall, our findings suggest that while normally developing vervets recognize the correspondence between the faces and voices of another species, those with FAE fail to do so. This finding raised a very important question regarding the domain-specificity of the observed deficit in vervets with FAE. That is, is the failure to integrate auditory and visual information restricted to voices and faces or does it affect sensory integration at a much broader scale to include other forms of auditory and visual stimuli? Our prediction was that given the extent of observed neuroanatomical abnormality in the frontal lobes of this model of FASD, we would observe a domain-specific deficit affecting only the integration of faces and voices. To test this hypothesis, we designed two additional experiments employing the same experimental design as that found in the first study.

**Figure 2:**
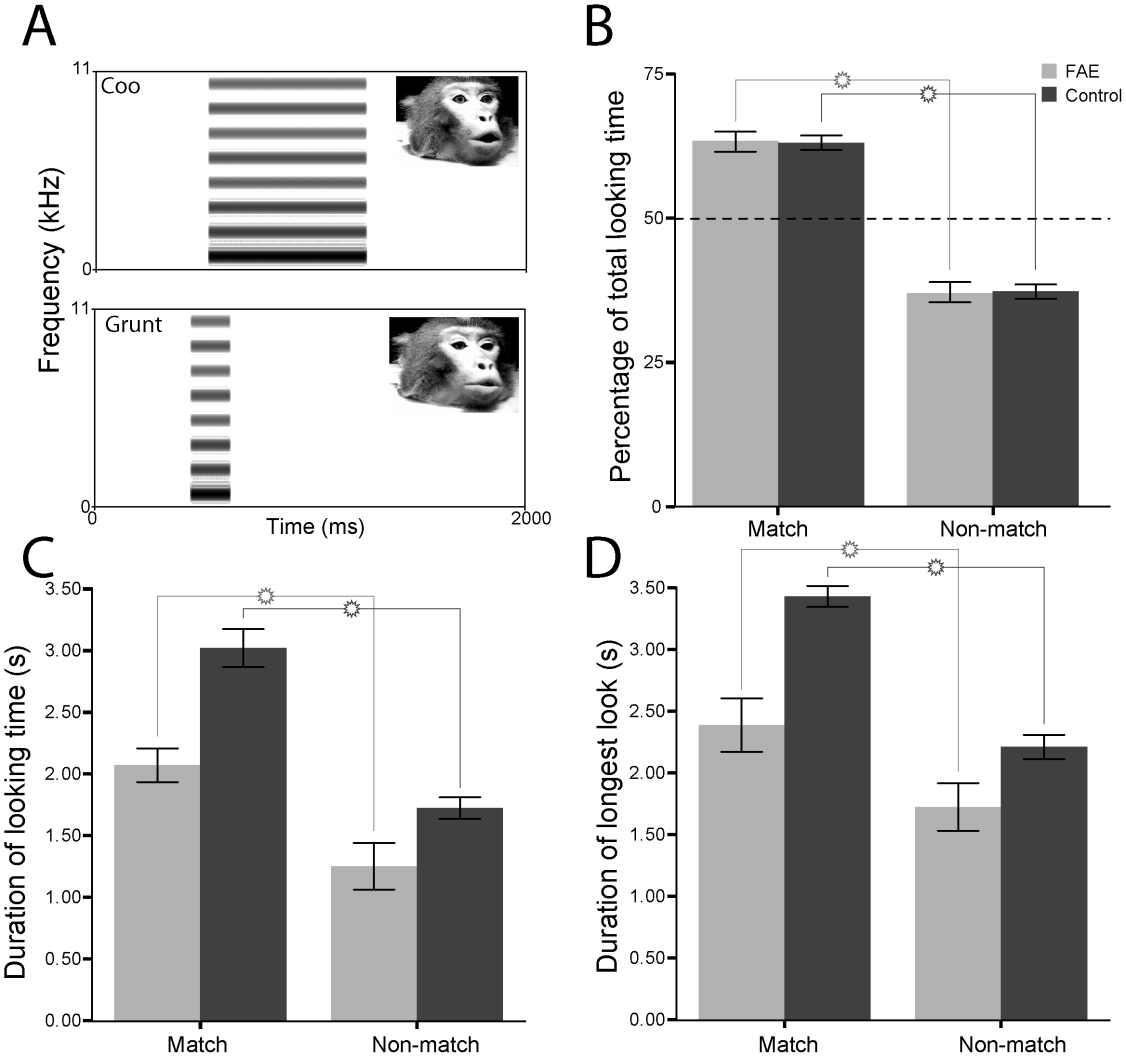
Looking behaviour of control (dark grey) and FAE (light grey) infant vervets at dynamic audiovisual presentations of rhesus monkey faces paired with complex tones in Experiment 2. A. Spectrograms of the tone stimuli showing that they were matched in duration to the original vocal sound track. The average fundamental frequency of the two vocalizations was used as the fundamental frequency for both complex tones. Inset shows one frame of the peak of the corresponding facial expression. B. The proportion of total looking time that the subjects spent looking at the matching face. C. The mean duration of looking time to the matching and nonmatching faces. D. The mean duration of the longest single look at the matching and nonmatching faces. Error bars represent ±1 SEM.

First, we tested another group of FAE (22–71 wks old) and control (22–50 wks old) vervets using the same visual stimuli, but with the vocalizations replaced by a complex tone that was broadband and that had the same duration and average fundamental frequency as the original vocalizations (Face + Complex Tone condition; Figure 2A). Importantly, the tone had a constant intensity and a linear spectral profile, and thus lacked the species-specific amplitude envelope and formants that are salient features of speech and of nonhuman primate vocalizations [55]–[58]. The results were consistent with our predictions. When we degraded the fine-grained spectrotemporal correlations (e.g., amplitude fluctuations) that the voice component bore with respect to the dynamic faces and, thus, reduced the overall salience of the stimulation, both the control and FAE vervets exhibited evidence of intersensory matching. Control and FAE subjects looked significantly longer both in terms of percentage of total looking time (p<0,001; Figure 2B) and mean duration of looking to the matching versus non-matching face (3.015±1.13 vs 1.719±0.648 seconds, p<0.001; Figure 2C).

In addition, the longest single looks were to the matching side for both the FAE (2.379±0.649 vs 1.718±0.573seconds; p = 0.003) and the control vervets (3.419±1.22 vs 2.204±0.696 seconds; p<0.001; Figure 2D). Together, these data represent one line of evidence that supports our hypothesis of domain-specificity of the auditory-visual integration deficits observed in the first group of FAE vervets. That is, the inability to match a facial expression to a vocalization does not appear to stem from a global deficit in matching corresponding auditory and visual stimuli but specifically from an inability to integrate corresponding faces and voices. As before, a repeated-measures ANOVA revealed no side, sound or face biases.

**Figure 3:**
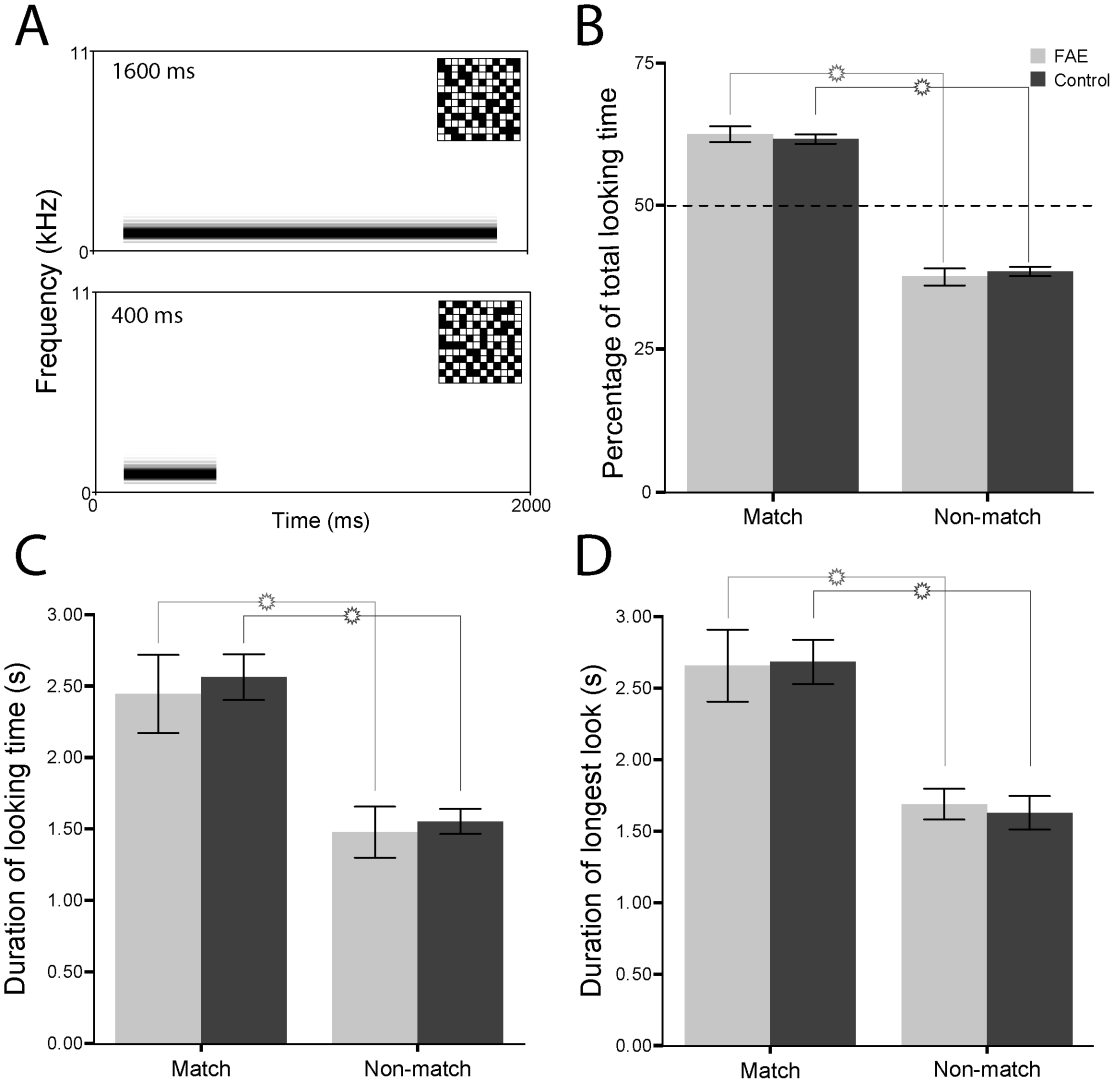
Looking behaviour of control (dark grey) and FAE (light grey) infant vervets at dynamic audiovisual presentations of random checkerboards paired with pure tones in Experiment 3. A. Spectrograms of the long and short duration pure tones. Inset shows one examples of corresponding checkerboards. B. The percentage of total looking time that the subjects spent looking at the matching checkerboard. C. The mean duration of looking time at the matching and nonmatching checkerboards. D. The mean duration of the longest single look at the matching and nonmatching checkerboards. Error bars represent ±1 SEM.

The results from the Face + Complex Tone condition suggest that the FAE vervets may be using low-level cues to detect the audiovisual correspondences. This constituted the second test of our hypothesis during which, we measured their looking behaviour towards an arbitrary combination of checkerboards and pure tones of two different durations (Checkerboard + Tone condition; Figure 3A). During the test, the onset and offset of the pure tone was synchronized to only one of the two checkerboards. Similar tests have been done with human infants [59]. In essence, there were side-by-side black-and-white checkerboards that were illuminated simultaneously but for different periods of time (e.g., 400 or 1600 ms). A pure tone of 1000 Hz was also presented at the onset of illumination. On each trial, the tone lasted as long as only one of the two checkerboards remained illuminated. The same two groups of control and FAE vervets, which were used in the Face + Complex Tone condition were tested with these stimuli. The results revealed that they could detect audiovisual correspondences between arbitrary stimuli using low-level temporal cues (duration and synchrony). The percentage of total looking time to the matching side was significantly greater than to the non-matching side for both FAE (p<0.001) and control vervets (p<0.001; Figure 3B). Similarly, the mean duration of looking to the matching checkerboards for both (p<0.001) and control vervets (p<0.001; Figure 3C) were significantly longer than to the non-matching checkerboard. Lastly, the average longest single look at the matching checkerboard was significantly longer than at the non-matching checkerboard for both FAE (2.648±0.251 vs 1.684±0.107 seconds; p = 0.006) and control vervets (2.674±0.155 vs 1.624±0.117 seconds; p<0.001; Figure 3D). These findings further support the hypothesis that FAE vervets exhibit a domain-specific deficit in the integration of voice and face stimuli, as opposed to a domain-general failure to integrate all corresponding auditory and visual stimuli.

Our third test of the hypothesis was to measure the relative amount of pupil dilation, both within conditions (match versus nonmatch) and across conditions (Face + Voice versus Face + Complex Tone). The rationale behind this test was based entirely on an earlier study showing that normally developing vervets have a pupillary response to macaque vocalizations, presumably due to anxiety and stress [41]. While pupils dilate in the dark and constrict in bright light, their diameter is also rapidly modulated by the valence of emotional stimuli or their ‘interest’ value [60], [61]. The most reliable pupillary response is dilation in response to unpleasant stimuli [60]. Thus, a strong prediction of our hypothesis is that while control vervets should have a greater pupillary response (in the form of dilation) while viewing matching rhesus monkey stimuli in the Face + Voice condition, this response should be absent in the FAE group. Furthermore, the matching versus nonmatching difference should be absent in the Face + Complex Tone condition for both groups.

**Figure 4:**
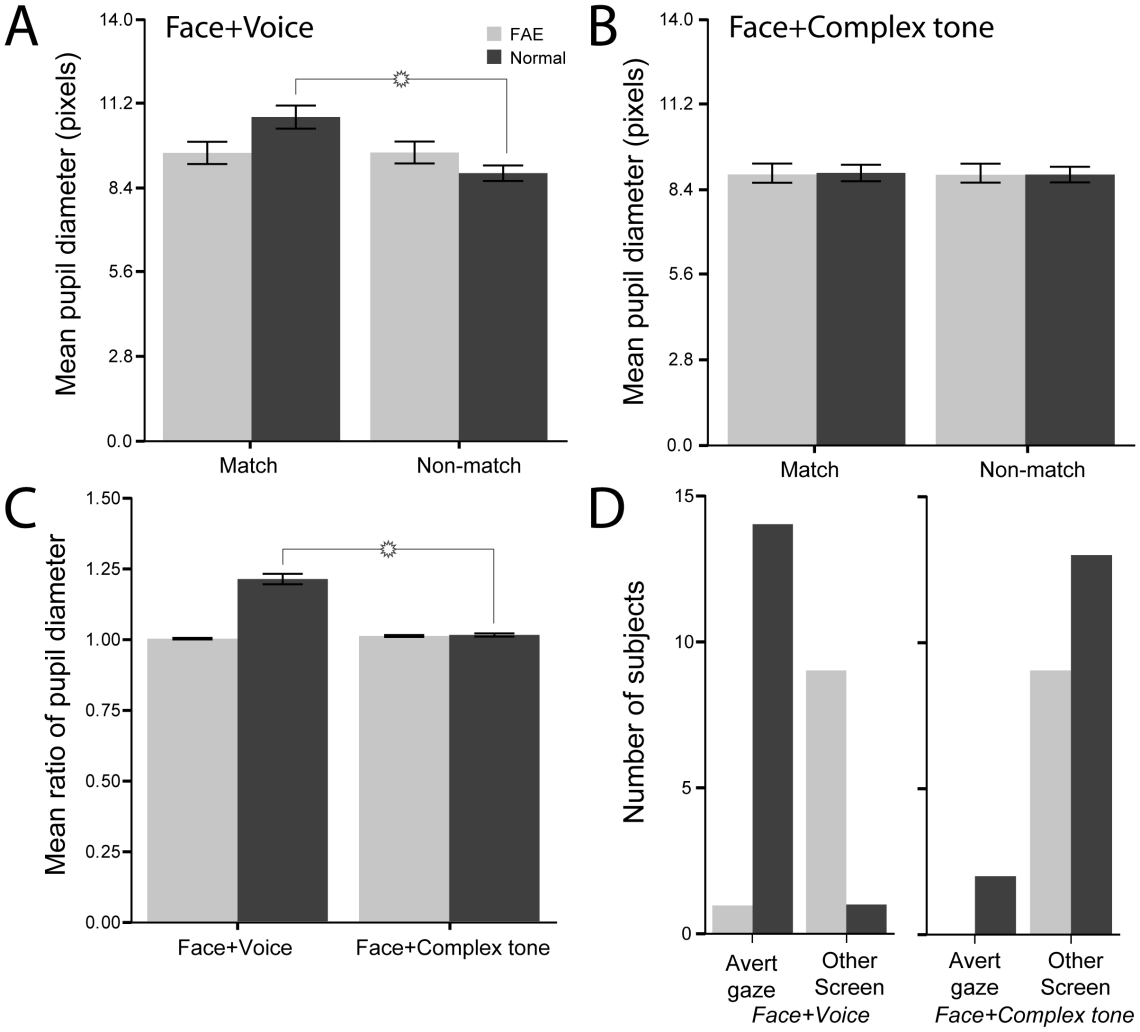
Pupillary response measures to each of the stimulus conditions. A. Mean pupil diameter (in pixels) at the end of the longest single look at the matched face versus mismatched face in the Face + Voice condition. B. Mean pupil diameter (in pixels) at the end of the longest single look at the matched face versus mismatched face in the Face + Complex Tone condition. C. The mean ratio of pupil dilation between matched versus mismatched looks across the two conditions. D. The proportion of subjects that averted their gaze versus those who looked at the other face following their single longest looks in the Face + Voice and Face + Complex Tone conditions. Error bars represent ±1 SEM.

We randomly selected 15 control subjects from each of the Face + Voice and the Face + Complex Tone conditions. For each subject, pupil and iris diameters were measured at the end of the ‘longest look’ towards the matching face and at the end of the longest look towards the nonmatching face (Figures 1D and 2D). Across our sample, the mean pupil diameter was significantly greater when control, but not FAE subjects were viewing the matching Face + Voice versus the nonmatching screen (10.823±0.392 vs. 8.988±0.252 pixels; p<0.001; Figure 3A). In contrast, in the Face + Complex Tone experiment, no such pupillary response differences were evident (9.19±0.28 vs. 9.14±0.252 pixels, p = 0.294; Figure 4B). Comparing the match/nonmatch ratio between the two conditions also revealed that pupil dilation is significantly greater in the Face + Voice versus Face + Complex Tone experiment (1.21±0.018 vs 1.01±0.006; p<0.0001; Figure 3C). These pupillary response data support our hypothesis in two ways. First, they show that while control vervet monkeys found the matching face to be more salient than the non-matching face in the presence of the natural voice, FAE vervets failed to exhibit this reflexive response. Second, they show that only control vervet monkeys, but not FAE counterparts, found the matching face in the presence of the natural voice to be more salient than the matching face in the presence of the complex tone. Importantly, this means that FAE vervets could not distinguish between the correspondence of a face and a natural voice, and the correspondence of the same face with a complex tone.

Finally, we tested the salience of the matching Face + Voice stimuli using a gaze-aversion measure. This measure provides another useful index of whether the vervets found the matching Face + Voice stimuli more arousing than the Face + Complex Tone stimuli. Using the same subjects per condition as in the pupillary response analysis, we scored whether at the end of their longest single looks directed at the matching Face + Voice versus the matching Face + Complex Tone stimuli, the vervets' first response was to avert their gaze from both faces (by looking away or closing their eyes) or to simply look at the other, nonmatching face. Figure 4D shows that for the Face + Voice condition, 14 out of 15 vervets averted their gaze (binomial test, p = 0.0001), while in the Face + Complex Tone condition, only 2 out of 15 vervets averted their gaze; the rest of the subjects looked toward the other face (binomial test, p = 0.007). These data also support the hypothesis that the failure of FAE vervets to integrate auditory and visual stimuli is specific to the domain of faces and voices.

## Discussion

The ability to adequately receive, process and adapt to environmental stimuli ultimately dictates how one perceives and interacts with one's environment. This is true not only of the physical environment, but also of the social environment. In dealing with the latter, affective cues are extremely important. The deficits in multisensory processing shown here in FAE, but not control, vervets may originate in the respective sensory-specific cortical areas, but the multimodal integration of the sensory information resides within the prefrontal cortex. Specifically the ventrolateral prefrontal cortex (vlPFC), which contains unimodal processing areas for both auditory and visual information, possesses neurons that integrate audiovisual communication stimuli [31], [34]. This circuit may be the neuroanatomical underpinning for the manifestation of the observed domain-specific deficit.

There are two converging lines of evidence that implicate frontal lobe structures in the manifestation of the observed deficit in FAE monkeys. First, a series of recent studies have investigated the multisensory capacities of the frontal lobes neurons, especially in the context of communication information processing. In particular, Sugihara et al [34] have elegantly documented the anatomical and functional properties of individual prefrontal cortical neurons in the integration of communication signals originating in the face and voice information. The results of their study suggest that those neurons form an important node in the cortical network responsible for communication. These data are thus congruent with neuroanatomical studies showing a paucity of frontal cortical neurons in FAE monkeys compared to age-matched normally developing conspecifics [39]. Together, these findings may help define the anatomical underpinnings of the observed domain-specific deficit in multisensory integration of faces and voices in FAE animals.

More importantly, there is ample evidence from the current study to suggest that the observed deficit is specific to the domain of face-voice integration and is not the result of a pan-sensory impairment affecting integration of auditory and visual information at all levels. Specifically, FAE animals fail to distinguish matching and non-matching faces in the context of a affectively salient macaque vocalization, while retaining a normal capacity to discriminate between a matching and a non-matching face in the context of a complex tone whose duration is the same as mouth opening. Further specificity is provided by the ability of FAE animals to discriminate between matching and non-matching lit checkerboards in the context of a pure tone. Therefore, one can conclude from these observations that FAE animals are capable of detecting and integrating the correspondences between auditory and visual stimuli based on low-level temporal cues (i.e., onset and offset synchrony, as well as duration). However, when the auditory stimulus conveys affective and social information, those animals fail to display looking behaviour that is suggestive of proper sensory integration. The pervasive social cognitive deficits documented in children with FASD may be due in part to a dysfunction in sensory integration similar to that shown here following naturalistic prenatal ethanol exposure in the non-human primate.
